# Identification of Corrosion Minerals Using Shortwave Infrared Hyperspectral Imaging

**DOI:** 10.3390/s22010407

**Published:** 2022-01-05

**Authors:** Thomas De Kerf, Georgios Pipintakos, Zohreh Zahiri, Steve Vanlanduit, Paul Scheunders

**Affiliations:** 1 Invilab Research Group, Faculty of Applied Engineering, University of Antwerp, 2020 Antwerp, Belgium; steve.vanlanduit@uantwerpen.be; 2 EMIB Research Group, Faculty of Applied Engineering, University of Antwerp, 2020 Antwerp, Belgium; Georgios.Pipintakos@uantwerpen.be; 3 Imec-Vision Lab, Physics Department, University of Antwerp, 2020 Antwerp, Belgium; zohreh.zahiri@uantwerpen.be (Z.Z.); paul.scheunders@uantwerpen.be (P.S.)

**Keywords:** hyperspectral imaging, FTIR, corrosion, shortwave infrared

## Abstract

In this study, we propose a new method to identify corrosion minerals in carbon steel using hyperspectral imaging (HSI) in the shortwave infrared range (900–1700 nm). Seven samples were artificially corroded using a neutral salt spray test and examined using a hyperspectral camera. A normalized cross-correlation algorithm is used to identify four different corrosion minerals (goethite, magnetite, lepidocrocite and hematite), using reference spectra. A Fourier Transform Infrared spectrometer (FTIR) analysis of the scraped corrosion powders was used as a ground truth to validate the results obtained by the hyperspectral camera. This comparison shows that the HSI technique effectively detects the dominant mineral present in the samples. In addition, HSI can also accurately predict the changes in mineral composition that occur over time.

## 1. Introduction

Corrosion is the destructive attack of a metal by reaction with the environment [1], especially with pollutants present in the air. The cost related to the deterioration of steel structures due to corrosion is estimated to be 3–4% of a nation’s GDP [2]. Therefore, proper detection and monitoring of corrosion products is necessary to reduce maintenance costs of steel structures. Several methods have been developed in the past to detect and/or quantify the presence of corrosion. Non-destructive techniques such as electrochemical impedance spectroscopy (EIS) [3], linear polarization resistance (LPR) [4], Ultrasonic measurement, Eddy current [5] and Electrical resistance (ER) [6] can measure the corrosion rate. These techniques are all single point measurements. It is challenging to extrapolate these single point measurements to an entire structure. Some non-destructive approaches such as thermography [7] or surface profile measurements [8] estimate the material loss occurring during corrosion.

A contrasting approach to assess the severity of a corrosion process is to gain a detailed understanding of the corrosion products that form on the surface. There are numerous approaches that can be used to identify various corrosion products, such as X-ray powder diffraction (XRD) [9,10,11,12,13], X-ray photoelectron spectroscopy (XPS) [14,15,16], scanning electron microscopy (SEM -EDS) [12,17], Fourier transform infrared spectroscopy (FTIR) [11,16,18,19,20,21], Raman Spectroscopy (RS) [22,23,24] and Mossbauer spectroscopy [11,21,24]. These methods can identify the types of oxides that can provide more information about the development of rust layers. This information leads to accurate predictions about the protective properties of the corrosion formed. For example, a protective ability index (PAI) for archaeological iron artifacts was proposed by Yamashita et al. [23] and developed into a corrosion stability value by Veneranda et al. [18] and Dillmann et al. [25]. This corrosion stability value is a ratio between stable corrosion compounds (goethite and magnetite) and corrosion-accelerating compounds (lepidocrocite and akagenite ). FTIR is a well-suited technique to distinguish the different iron oxides. Each mineral can be identified by multiple distinct peaks in the spectrum, as demonstrated in [20,26]. Compared to other techniques, FTIR is easy to use and the spectral analysis is straightforward. A list of the governing functional groups alongside with their peaks ascribed to different corrosion products have been published in several papers, a review can be found in [26].

However, the small sample size, fragility and size of FTIR measurement equipment imply that FTIR analysis must be carried out in a laboratory environment. For industrial or in situ use, a hyperspectral camera is much more suitable. The acquisition time is fast (it can be as fast as 20 mm/s for line scan devices, depending on the FOV used) and the device is portable. The main advantage is that a HSI technique can be used to scan an entire structure, thus eliminating the need to extrapolate single point measurements.

There have been a limited number of studies that use hyperspectral imaging to detect corrosion. For instance, Halford et al. [27] used a HSI to inspect corrosion on bronze sculptures. As for steel samples, Antony et al. [28] used an HSI with a fiber bundle probe attached to classify corrosion on steel samples. Thanks to the fiber bundle, the device can be efficiently used in difficult to access spaces. In the paper, a classification is done between bare metal, corrosion and cladding. However, in both papers, the mineral composition is not investigated.

As mentioned before, the knowledge of the type of corrosion mineral present can be of great importance for characterizing the corrosion process. This article aims to combine the ability of measuring corrosion products along with the ability to scan an entire structure by using a hyperspectral camera to detect corrosion minerals on carbon steel samples.

The manuscript is structured as follows. In Section 2, the experimental data are described, the FTIR semi-quantitative analysis method is disclosed and the hyperspectral measurement setup together with the analysis methods are introduced. This is followed by Section 3, where first, the results of the FTIR analysis are explained. The FTIR analysis is then used as a ground truth to evaluate the accuracy of the HSI method. In Section 4, the conclusion is presented and possible further investigations are proposed.

## 2. Material and Methods

### 2.1. Corrosion Samples Preparation

**Figure 1:**
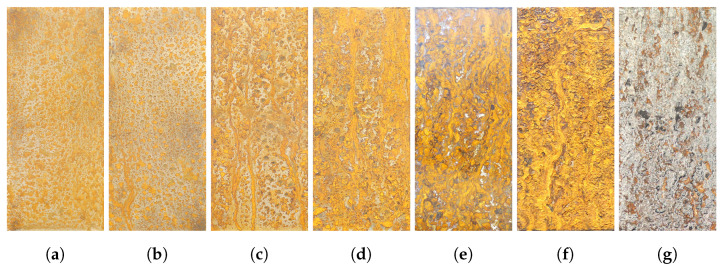
RGB images of the different samples. (**a**) Sample 1; (**b**) Sample 2; (**c**) Sample 3; (**d**) Sample 4; (**e**) Sample 5; (**f**) Sample 6; (**g**) Sample 7.

In order to obtain samples with different corrosion mineral composition, seven corrosion samples of carbon steel (s235) were prepared. The steel specimens with dimensions of 150 mm × 50 mm × 10 mm were milled to remove mill scale and then manually abraded with 400 grit sandpaper to obtain the same surface roughness for all specimens. After cleaning with a degreaser, the specimens were placed in a salt spray chamber. To accelerate the corrosion process, the specimens were exposed to a neutral salt spray test. During this test, the pH value of the sprayed solution was kept between 6.8 and 7.1 and the operation temperature was kept at 35 °C. These parameters are in accordance with DIN ISO 9227 [29]. The test duration varied from 30 min to 72 h, see Table 1 for a summary of duration and sample number. To create a difference in corrosion mineral composition, the salt spray was turned off after Sample 6 was removed from the salt spray cabinet. Thus, Sample 7 was subjected to a drying process for 24 h. Figure 1 shows the RGB images of the samples acquired with a digital single lens reflex camera (SLR). What stands out in this figure is the similarity in color between Sample 1 through 6; the same brown orange color can be observed in all of the samples. As for Sample 7, seen in Figure 1g, the color changes from orange/brown to gray.

To obtain the corrosion products from the samples, a clean metal knife is used to scrape the corrosion products from the samples. For each sample, the entire sample surface was scraped.

### 2.2. FTIR Measurements

To obtain spectra from the corrosion powders, a benchtop Scientific Nicolet iS10 FTIR spectrometer equipped with an Attenuated Total Reflectance (ATR) fixture (diamond crystal) and a Smart Orbit Sampling Accessory was utilized. For the acquisition of the spectra, 5 equivalent sample replicates were recorded in absorbance mode in a wavelength range varying from 400 ${\mathrm{cm}}^{-1}$ to 4000 ${\mathrm{cm}}^{-1}$. The resolution was fixed at 4 ${\mathrm{cm}}^{-1}$ and the spectra were acquired as the average of 32 repetitive scans. Five equivalent measurements were performed on each sample. The spectra were further analyzed using python.

### 2.3. Semi-Quantification of Corrosion Products from FTIR

**Figure 2:**
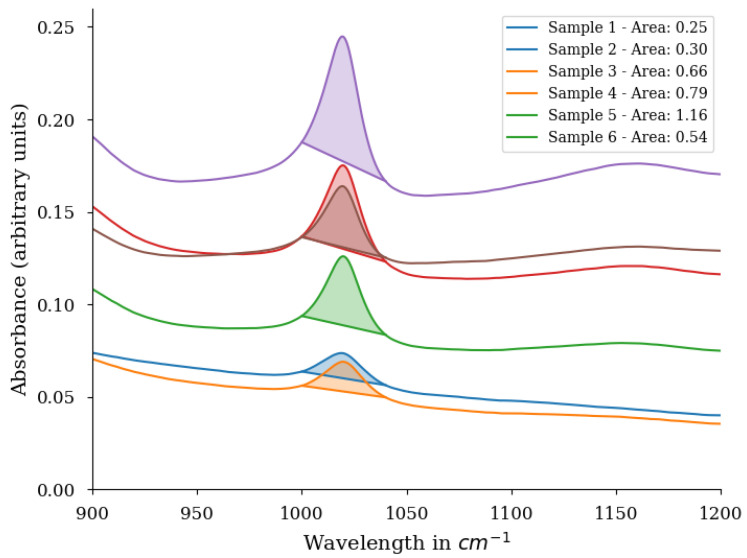
Example of Area under the curve (AUC) calculations for Fourier Transfor Infrared Spectroscopy (FTIR) measurements. The specific peak shows a Lepidocrocite peak at 1100 ${\mathrm{cm}}^{-1}$. Sample 7 is excluded from this graph to improve the readability.

FTIR measurements have been used as a quantitative technique in previous research: [18,20,30,31]. In this article, a combination of the work of [30,31] is used to semi-quantify the corrosion products. First, a normalization is performed on the data [31]:

(1) $$N_{i}=\frac{x_{i}-I_{min}}{I_{max}-I_{min}}$$

where $N_{i},x_{i},I_{max}$ and $I_{min}$ stand for the normalized, absolute, maximum absolute and minimum absolute intensities of a single spectrum, respectively. The second step is to define the peak in the spectrum that corresponds to the corrosion minerals and calculate the area under the curve (AUC) [31]. This is shown in Figure 2. After this step, a matrix is obtained that lists the area size of each mineral and this for each sample. To obtain the semi-quantification mineral abundance per sample, there are two more steps required. The first step is a normalization per mineral from 0 to 1 over all the samples. This means that the calculated area size for an individual mineral that has the lowest value in a sample, is 0 and the area size that is the largest is 1 for a different sample. The last step is to normalize over each sample. For example, if you have two minerals in the sample with 0.1 and 0.4 as normalized AUC, then we normalize these values so that the sum of those values is equal to 1 (the resulting values would be 0.2 and 0.8).

This results in a mineral composition that serves as the ground truth, to evaluate the HSI measurements.

### 2.4. Hyperspectral Measurements

The hyperspectral camera used in this study is a Specim FX17 instrument operating in the SWIR range (900 nm to 1700 nm). The instrument operates as a line scan camera, with each line containing 640 pixels and measuring 224 bands for each pixel with a full width at half maximum (FWHM) of 8 nm. A lens with a field of view of 56° is used with a standoff distance of 32 cm, resulting in a spatial resolution of 0.1 mm/pixel. To calibrate the images a dark reference (closing the shutter) and a white reference measurement was done. A calibrated spectralon tile with 99% reflectance was used for the white reference measurement.

### 2.5. Hyperspectral Classification Algorithm

**Figure 3:**
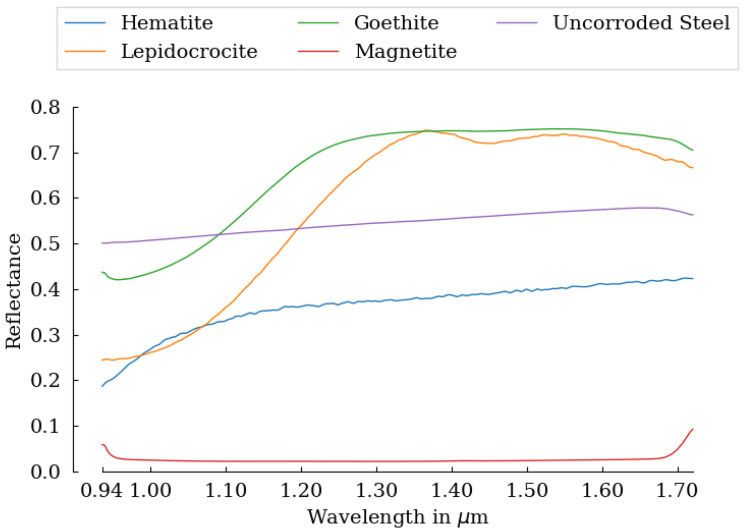
Spectra of the different corrosion minerals found in the USGS spectral library [32]. Uncorroded Steel is an own measurement before the samples underwent corrosion.

To classify each pixel of the HSI, the spectra are correlated to reference endmember spectra, i.e., pure spectra of the minerals under investigation. The reference spectra were obtained from in [32] and are displayed in Figure 3. For some spectra, there are multiple spectra in the database that differ in mineral purity and spectrometer used. In this article, the measurement with the highest purity, purity code a, is used as a reference for that mineral. Purity code a indicates that, based on other methods (for example, XRD or microscopic examination), no contaminants are present Kokaly et al. [32].

To correlate the measured spectra to the references, a normalized cross-correlation algorithm is used. The matching is achieved by subtracting the mean and dividing by the standard deviation:

(2) $$NormCorr=\frac{1}{N}\sum \frac{\left(s_{r}-\mu_{s_{r}}\right)\left(s_{m}-\mu_{s_{m}}\right)}{\sigma_{s_{r}}\sigma_{s_{m}}}$$

where $s_{m}$, $s_{r}$ are the measured and reference spectra, $\mu_{s_{m}}$ and $\mu_{s_{r}}$ are the mean of the measured and reference spectra, and $\sigma_{s_{m}}$ and $\sigma_{s_{r}}$ are the standard deviations of the measured and reference spectra. The result of the cross-correlation algorithm (NormCorr) is a value between −1 and 1, where 1 means a perfect match with the reference spectrum (endmember). Each pixel is cross-correlated with the 5 reference spectra and the highest value is then selected as the corresponding class. A threshold value of 10% is used to filter out pixels that do not show any correlation between the different minerals, these pixels are then classified as ‘Unknown’.

## 3. Results

The results are presented in three sections. Section 3.1 shows the results of the FTIR measurements on the scraped corrosion sample powders and discusses the presence and identification of the different corrosion minerals. These results act as the ground truth for the second part, Section 3.2. In this second part, the results of the HSI measurements of the full corrosion samples are presented and the classification maps are shown and discussed in detail. Section 3.3 compares the semi-quantification results of the FTIR measurement and the classification efforts on the HSI.

### 3.1. FTIR Measurements of Corrosion Powders

**Figure 4:**
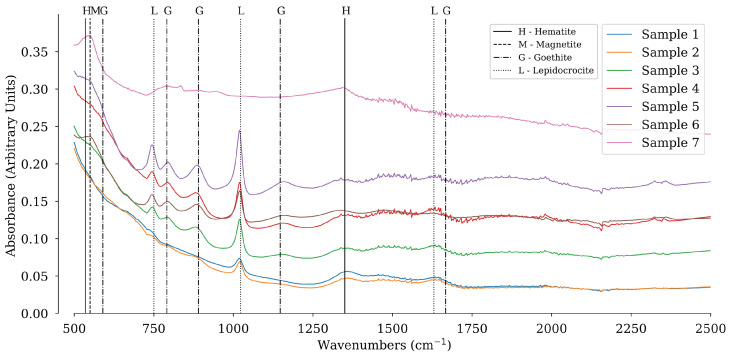
FTIR spectra of all samples. The characteristic wavenumbers for each corrosion mineral are indicated by vertical lines, with an abbreviation for each mineral above the vertical line. The colored lines represent the spectra of the different samples.

Figure 4 shows the FTIR measurements. The vertical colored lines represent the peaks for the different corrosion minerals as found in the literature. Lepidocrocite (blue) is well represented in Samples 1 to 6 with 2 distinct peaks of around 750 and 1023 ${\mathrm{cm}}^{-1}$. The peak at 1023 ${\mathrm{cm}}^{-1}$ increases from Samples 1 to 5 and decreases again in Sample 6 to the level of Sample 4. This indicates that the proportion of lepidocrocite increases from Samples 1 to 5, decreases in Sample 6 and disappears completely in Sample 7. Goethite does not appear to be present in Samples 1 and 2. From Samples 3–5 the specific peaks at 791 and 890 ${\mathrm{cm}}^{-1}$ increase in height. From Sample 6, the goethite peaks decrease to a similar level as in Sample 3, and in Sample 7 the peaks disappear completely, indicating that goethite is no longer present.

### 3.2. Hyperspectral Imaging Samples

**Figure 5:**
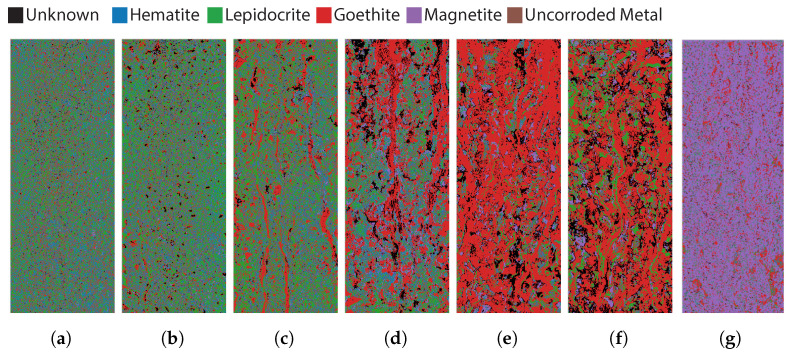
Classification results based on the Normalized Cross Correlation of the different samples. (**a**) Sample 1; (**b**) Sample 2; (**c**) Sample 3; (**d**) Sample 4; (**e**) Sample 5; (**f**) Sample 6; (**g**) Sample 7.

Figure 5 shows the classification results of the hyperspectral measurements using the normalized cross-correlation algorithm. Note in this figure that there is a transition from lepidocrocite (green) to goethite (red) and finally magnetite (purple). There is also a significant amount of uncorroded metal in the first two images, which is a correct analysis when compared to the RGB images in Figure 1a,b. Samples 1 and 2 are in a very early stage of corrosion and are not completely covered with corrosion products. In Sample 3, goethite appears on the sides and in larger streaks. In Sample 4, this increase in goethite continues, and in Sample 5 the whole sample is predominantly classified as goethite. In Sample 6, more goethite is again noted along with lepidocrite, but the dominant mineral is still goethite. Sample 7 is almost entirely composed of magnetite. This sudden transition from goethite to magnetite can be atrributed to the drying process between Samples 6 and 7, which is confirmed by the FTIR measurement of Sample 7.

### 3.3. Comparison between FTIR and Hyperspectral Imaging (HSI)

**Figure 6:**
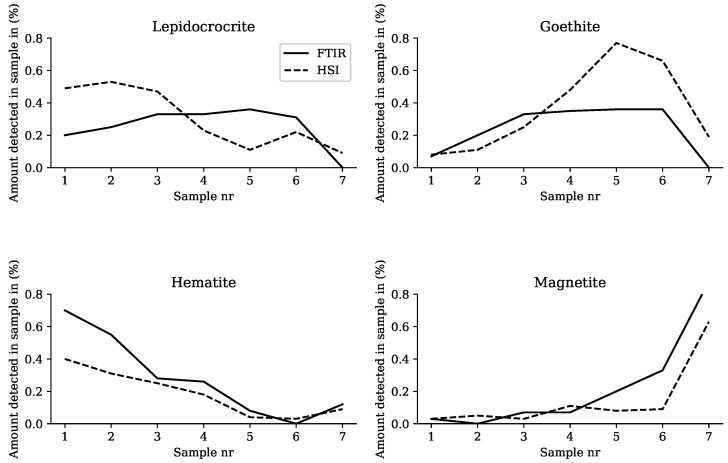
Comparison between the mineral quantities of the FTIR and HSI measurements.

For the FTIR measurements on the scraped powder from the samples, each mineral quantity can be expressed as a percentage of the total. This is implemented by using the semi-quantitative technique, described in Section 2.3. As for the HSI measurements on the entire corroded samples, the mineral quantity can also be expressed as a percentage of the total. This is carried out by counting the amount of classified pixels for each corrosion mineral. The comparison between the mineral quantities of the FTIR and HSI measurements is shown in Figure 6. Each mineral is shown on a separate graph, with the sample number on the x-axis and the percentage on the y-axis.

When considering lepidocrocite, the FTIR measurement shows that the mineral is present in the corrosion powder and increases steadily from Samples 1 to 4, then decreases from Sample 5. This trend is not reflected in the measurement from HSI. What is noticeable is that in Sample 5, there is an overclassification of goethite at the expense of lepidocrocite. Looking at Figure 3, it appears that the spectra of goethite and lepidocrocite are quite similar in the SWIR region. This could explain the large deviation between the HSI and FTIR measurements in Sample 5. Another possible reason for this deviation could be that the depth of the scraping has an influence. The corrosion products consist of layers of different corrosion minerals which could be difficult to detect using HSI, as HSI is only able to detect surface defects. Apart from this deviation in Sample 5, the measurements for goethite are quite similar.

The FTIR and HSI results for hematite and magnetite show a very similar pattern. However, the largest absolute error is still 36% and 31% between the hematite and magnetite measurements respectively.

## 4. Conclusions

This study set out to determine if HSI could be used to identify corrosion minerals present in carbon steel samples. From the comparison in Section 3.3, it can be concluded that HSI can be used to determine the dominant mineral present in the sample. The second finding is that HSI can be used to monitor general changes over time in the composition of corrosion minerals in carbon steel samples. This can be of interest for the monitoring of steel structures in a cost effective and automated manner using hyperspectral cameras, as the type of mineral indicates whether the corrosion process is active or dormant.

More research is required to increase the accuracy of mineral classification in the powders and samples using hyperspectral cameras. One way could be to apply spectral mixture analysis. Another way to increase the accuracy is to broaden the spectral range, e.g., by including the long wavelength infrared region, as the FTIR measurements show that this region contains unique spectral features for the corrosion minerals.

## Figures and Tables

**Table 1:** Overview of samples number and corresponding duration of the accelerated corrosion test.

Sample Nr	Duration (h)
1	0.5
2	1
3	4
4	8
5	24
6	48
7	72

## Data Availability

The data presented in this study are available on request from the corresponding author.

## Abbreviations

The following abbreviations are used in this manuscript:

HSI	Hyperspectral Imaging
FTIR	Fourier Transform Infrared Spectroscopy
GDP	Gross Domestic Product
EIS	Electrochemical Impedance Spectroscopy
LPR	Linear Polarization Resistance
ER	Electrical Resistance
XRD	X-ray diffraction
XPS	X-ray photoelectron spectroscopy
SEM-EDS	Scanning Electron Microscopy
AUC	Area Under the Curve
Norm Corr	Normalized Cross-Correlation
